# Oral exposure to commercially available coal tar‐based pavement sealcoat induces murine genetic damage and mutations

**DOI:** 10.1002/em.22032

**Published:** 2016-07-30

**Authors:** Alexandra S. Long, Margaret Watson, Volker M. Arlt, Paul A. White

**Affiliations:** ^1^ Department of BiologyUniversity of OttawaOttawaOntarioCanada; ^2^ Mechanistic Studies DivisionEnvironmental Health Science and Research Bureau, Environmental and Radiation Health Sciences Directorate, HECSB, Health CanadaOttawaOntarioCanada; ^3^ Analytical and Environmental Sciences DivisionMRC‐PHE Centre for Environment and Health, King's College LondonLondonUnited Kingdom

**Keywords:** coal tar, PAH, BaP, sealcoat, driveway sealant, genotoxicity

## Abstract

Coal tar (CT) is a thick black liquid produced as a by‐product of coal carbonization to produce coke or manufactured gas. It is comprised a complex mixture of polycyclic aromatic compounds, including a wide range of polycyclic aromatic hydrocarbons (PAHs), many of which are genotoxic and carcinogenic. CT is used in some pavement sealants (also known as sealcoat), which are applied to pavement in order to seal and beautify the surface. Human exposure is known to occur not only during application, but also as a result of the weathering process, as elevated levels of PAHs have been found in settled house dust in residences adjacent to CT‐sealed surfaces. In this study we examined the genotoxicity of an extract of a commercially available CT‐based sealcoat in the transgenic Muta™Mouse model. Mice were orally exposed to 3 doses of sealcoat extract daily for 28 days. We evaluated genotoxicity by examining: (1) stable DNA adducts and (2) *lacZ* mutations in bone marrow, liver, lung, small intestine, and glandular stomach, as well as (3) micronucleated red blood cells. Significant increases were seen for each endpoint and in all tissues. The potency of the response differed across tissues, with the highest frequency of adducts occurring in liver and lung, and the highest frequency of mutations occurring in small intestine. The results of this study are the first demonstration of mammalian genotoxicity following exposure to CT‐containing pavement sealcoat. This work provides *in vivo* evidence to support the contention that there may be adverse health effects in mammals, and potentially in humans, from exposure to coal tar. Environ. Mol. Mutagen. 57:535–545, 2016. © 2016 Her Majesty the Queen in Right of Canada

## INTRODUCTION

Driveway sealants (i.e., sealcoats) are widely used across North America by homeowners and commercial property owners to beautify and protect asphalt and concrete surfaces. There are two main formulations of sealcoat products used in North America: aqueous emulsions containing 10‐35% coal tar (CT) and aqueous emulsions containing a similar level of asphalt (i.e., bitumen) [Mahler et al., 2005]. Crude CT used in the production of the former is a thick black liquid, generated as a by‐product of coal carbonization to produce coke or manufactured gas. CT‐based sealcoats generally contain a refined material known as RT‐12 (i.e., ASTM D490, Road Tar 12), which is derived from high‐temperature CT pitch, the material that remains after removal of distillation products such as light and medium oils from crude CT. RT‐12 is the most viscous of 12 refined products derived from high‐temperature CT pitch.

CT is well recognized as a multi‐organ carcinogen in experimental animals [Robinson et al., 1987; Culp et al., 1998; Goldstein et al., 1998], and CT and CT pitch are both known human carcinogens [IARC, 1985; IARC, 2010a,b]. They contain a complex mixture of polycyclic aromatic compounds (PACs), including a wide range of polycyclic aromatic hydrocarbons (PAHs), many of which have the ability to damage DNA (i.e., are genotoxic), as well as cause mutations and cancer [IARC, 1983, 2010b]. In contrast, asphalt (‘bitumen’ in Europe), which is similar in appearance to CT, is a by‐product of petroleum refining and contains only low concentrations of genotoxic and carcinogenic PAHs. In addition, according to IARC's 2011 evaluation, there is only limited evidence that bitumens are animal carcinogens [IARC, 2011]. The concentration of priority PAHs (i.e., the 16 PAHs designated by the US EPA as priorities for concern and control [Keith and Telliard, 1979]) in CT‐based sealcoats is more than 1000‐fold greater than in asphalt‐based products [Mahler et al., 2012]. Although usage data for CT‐based sealcoats in Canada are not available, it is estimated that 320 million litres of CT‐based sealcoat are used annually in the United States (US) [Scoggins et al., 2009]. An estimated 82 to 100 kilotonnes of CT pitch is produced annually in Canada (unpublished data).

Sealcoat is applied to surfaces by either rolling/spreading or spraying. Human exposure is known to occur during the application process as well as from volatilization while drying. Subsequent exposure to particulate material can also occur from surface weathering. Two hours following product application, volatile PAH concentrations in air sampled at 0.03 m above CT‐sealed lots are 5000‐fold greater than those above unsealed lots [Van Metre et al., 2012]. In addition, PAH concentrations in water runoff from CT‐sealed lots were shown to be ∼65‐fold higher than in runoff from asphalt‐sealed lots, and a study of 40 US lakes determined that CT sealcoat was likely the greatest contributor to elevated levels of sediment PAHs [Mahler et al., 2005; Van Metre and Mahler, 2010]. Finally, PAH levels in settled house dust (i.e., SHD) collected from apartments adjacent to CT‐sealed lots were found to be 25‐fold higher than SHD samples from apartments adjacent to asphalt‐sealed lots, a difference that has been translated into significant elevations in excess lifetime cancer risk [Mahler et al., 2010; Williams et al., 2013]. A ban on CT‐based pavement sealcoats was implemented in the city of Austin, TX in November, 2005, and subsequent analysis of priority PAH levels in dated lake sediment cores showed a 44% decline in mean levels from 1998–2005 to 2006–2012 [Van Metre and Mahler, 2014]. Recently, in an effort to reduce PAH loading to local waterways via storm water discharge, other municipalities (e.g., Washington, DC) have implemented similar bans [District of Columbia, 2013].

The toxicity of CT‐based sealcoats and runoff has been demonstrated in several aquatic organisms. Studies of *Ceriodaphnia dubia*, *Pimephales promelas* [Mahler et al., 2015] and *Xenopus laevis* [Bryer et al., 2006] exposed to CT‐sealcoat runoff, and freshwater macroinvertebrates [Bryer et al., 2010] exposed to CT‐sealcoat in soil showed increased mortality relative to controls. Eastern newts exposed to CT‐sealcoat in sediment also demonstrated signs of toxicity [Bryer et al., 2010]. Additionally, a study by Kienzler et al. [2015] that employed the comet assay, showed increased DNA damage in a piscine liver cell line exposed to diluted CT sealcoat runoff.

Collectively, the aforementioned studies and reviews indicate that crude CT is mutagenic and carcinogenic, and known to contain a wide range of mutagenic and carcinogenic PAHs. Moreover, emissions from pavements treated with CT‐based sealcoat products, which can contribute to elevated levels of PAHs in air, SHD, soil, and aquatic sediments, are toxic and/or genotoxic to a variety of organisms. As such, CT‐based sealcoats constitute an environmental and human health hazard. However, although the genetic toxicity and carcinogenicity of CT and PAHs have been well documented [IARC, 1983; Culp et al., 1998; IARC, 2010b], the mammalian genotoxicity of commercially available CT‐based sealcoats has hitherto not been investigated.

In the current study, we have used the Muta™Mouse system to examine the genetic toxicity of an extract of a commercially available CT‐based sealcoat. We have previously demonstrated the ability to integrate several genotoxicity endpoints into a single *in vivo* murine study by employing the transgenic Muta™Mouse system [Lemieux et al., 2011]. Additionally, we have shown the reliability of the study design to detect induced mutations in multiple Muta™Mouse tissues following oral exposures to several PAHs [Long et al., 2016]. In addition, we have employed an *in vitro* version of the Muta™Mouse mutagenicity assay to assess the mutagenic activity of complex PAH‐containing extracts from CT‐contaminated soils [Lemieux et al., 2015a, 2015b]. Herein we report the genotoxicity observed in mice as a result of daily exposure to an extract of CT‐based sealcoat, including the frequency of stable DNA adducts and mutations found in bone marrow, liver, lung, small intestine, and glandular stomach, as well as the frequency of chromosome damage in peripheral red blood cells.

## MATERIALS AND METHODS

### Driveway Sealcoat Handling and Extraction

All chemicals used for the extraction were analytical grade and obtained from EMD Chemicals (Gibbstown, NJ, USA). A 15‐L pail of CT‐based sealcoat was purchased from a local home improvement retailer. The product is a water‐based CT emulsion that, according to the Material Safety Data Sheet for the product, contains 10–30% high‐temperature CT pitch (CAS # 65996‐93‐2) by weight. The sealcoat was thoroughly homogenized and aliquots of the homogenized material were dispensed on to several large glass petri dishes to air dry for 48 h. This drying process resulted in a total dry weight of 25% of the bulk sealcoat (i.e., water content ∼75%). Approximately 10 g of dry sealcoat was then scraped from the surface of the petri dishes, weighed on an analytical balance, and transferred to a coffee grinder, where it was ground into a fine powder. The resulting powder was combined with 25 g of attapulgus clay (Forcoven Products, Humble, TX), and extracted using a method adapted from Wise et al. [1988] that permits removal of solvent‐insoluble hydrocarbons (e.g., asphaltenes) and isolation of the complex, PAH‐containing fraction for chemical and toxicological analyses. Briefly, the sealcoat/clay mixtures were applied to three 10 cm diameter open columns each packed with 300 g of attapulgus clay conditioned with 10% dichloromethane (DCM) in pentane prior to the extraction. The following amount of dried CT sealcoat was applied to each column: (a) 10.47 g, (b) 10.11 g, and (c) 10.05 g. The columns were eluted with 1.8 l of 10% DCM in pentane, and the eluate was combined into a single vial and evaporated under ultrapure nitrogen until a viscous dark brown liquid remained that could no longer be reduced. The final volume of the sealcoat eluate was 5.85 ml. A small aliquot (100 µl) was removed for chemical analyses, and the remainder used to prepare dosing solutions by first sonicating the eluate and then diluting the material in an appropriate quantity of highly refined olive oil (Sigma‐Aldrich Canada, Oakville, ON). Doses were prepared based on the mg equivalent (eq) weight of the crude (i.e., wet) sealcoat.

### Chemical Analysis by GC–MS

The PAH content of the sealcoat concentrate was determined using GC–MS according to US EPA Method 8270D. The analyses were conducted by a commercial laboratory accredited by the Canadian Association for Laboratory Accreditation (CALA), the National Institute of Standards and Technology, and the National Voluntary Laboratory Accreditation Program. Briefly, the samples were warmed and diluted with DCM and an aliquot was analyzed on an Agilent 6890N gas chromatograph with a split/splittless injector and Restek Rxi‐5Sil MS column (30 mm × 0.25 mm ID, film thickness 0.25 μm, Chromatographic Specialties, Brockville, ON). Target analytes were identified and quantified using an Agilent 5973N mass spectrometer operating in SIM (selected ion monitoring) mode. The concentrations of a standard panel of 19 PAHs were determined and results expressed as mg PAH/kg sealant‐eq. This includes the 16 priority PAHs, as well as seven PAHs designated by the US EPA as class B2 carcinogens (i.e., probable human carcinogens) [USEPA, 1993].

### Animal Treatment

Adult Muta™Mouse (strain 40.6) males (18‐weeks of age) were randomly assigned to a dose group and individually housed in microVENT ventilated racks (Allentown, Allentown, NJ). The mice received standard rodent chow (2014 Teklad Global standard rodent diet) and water *ad libitum* for the duration of the study, and were maintained on a 12‐h light/12‐h dark cycle. The dosing solution was administered at 0.005 ml/g body weight (BW). The sealcoat extract was dissolved in highly refined olive oil and prepared at the following doses: 1974, 3949, 7897 mg crude sealcoat eq/kg BW/day. There were five mice in each dose group, including the control group (20 animals in total). The sealcoat extract was administered via oral gavage daily for 28‐days, followed by a 3‐day sampling time (i.e., no additional doses) prior to necropsy, according to OECD test guideline # 488 [OECD, 2011]. Two days after the final dose, blood was obtained from the facial vein for micronucleus frequency determination. Animals were euthanized via cardiac puncture under isofluorane gas anaesthesia, followed by cervical dislocation and chest cavity opening. The glandular stomach and small intestine were rinsed with phosphate‐buffered saline (PBS) (Thermo‐Fisher, Waltham, MA) to remove partially digested food and flash frozen along with liver and lung. The bone marrow was flushed from both femurs with PBS, pelleted, and flash frozen. All tissues were stored at −80°C. Mice were bred, maintained, and treated in accordance with the Canadian Council for Animal Care Guidelines and the protocols were approved by the Health Canada Ottawa Animal Care Committee.

### DNA Extraction

Murine tissues were prepared for genomic DNA extraction as described in Long et al. [2016]. Briefly, prepared tissues were suspended in freshly prepared ice‐cold lysis buffer [1 mM Na_2_EDTA, 100 mM NaCl, 20 mM Tris–HCl, pH 7.4, 1% SDS (w/v)] and incubated overnight at 37°C with gentle shaking. Genomic DNA was isolated from lysed tissues using a phenol/chloroform extraction procedure described previously [Douglas et al., 1994; Vijg and Douglas, 1996]. Isolated DNA was dissolved in 100 µl TE buffer (10 mM Tris pH 7.6, 1 mM EDTA) and stored at 4°C until use.

### Analysis of DNA Adducts by ^32^P‐Postlabeling

The nuclease P1 enrichment version of the thin‐layer chromatography (TLC) ^32^P‐postlabeling assay was used to determine DNA adduct formation in bone marrow, liver, lung, glandular stomach, and small intestine. The procedure was performed as described previously [Wohak et al., 2014; Krais et al., 2015]. As in prior studies [Kim et al., 2011; Siddens et al., 2012; Longhin et al., 2013; Molina et al., 2013], total DNA adduct levels were measured in the diagonal radioactive zone (DRZ) area of the TLC plates and were considered representative of PAH–DNA and other aromatic/hydrophobic adducts resistant to nuclease P1 digestion. The method provides a summary measure of a complex mixture of adducts present in the postlabeling chromatograms. The results were expressed as DNA adducts/10^8^ nucleotides.

### Analysis of *lacZ* Mutations by the PGal Positive Selection Assay

The PGal (phenyl‐β‐d‐galactoside) positive selection assay was used for the determination of *lacZ* mutant frequency in DNA samples from bone marrow, liver, lung, glandular stomach, and small intestine, as previously described [Gossen et al., 1992; Vijg and Douglas, 1996; Lambert et al., 2005]. Mutant frequency was calculated as the ratio of mutant plaque forming units (i.e., pfu) to total pfu.

### Analysis of Peripheral Blood Micronuclei by Flow Cytometry

MicroFlow^®^ kits (Litron Laboratories, Rochester, NY) were used for enumeration of micronucleated reticulocytes (RET) and normochromatic erythrocytes (NCE). Briefly, approximately 60 µl of peripheral blood was immediately combined with 350 µl of anticoagulant. Blood samples were then fixed by transferring to ice‐cold methanol and stored at −80°C. After 3–5 days, fixed blood samples were then centrifuged, rinsed, and transferred to a long‐term storage solution. Coded specimens were shipped to Litron Laboratories (Rochester, NY) for analysis. Micronuclei were scored in NCEs (i.e., MN‐NCEs) and RETs (i.e., MN‐RETs) by flow cytometry using a 3‐color labeling method described in Torous et al. [2001] and Dertinger et al. [2004].

### Statistical Analysis

The *lacZ* and MN dose–response data were analyzed in SAS v.9.1 (SAS Institute, Cary, NC) by Poisson regression and a Type 3 chi‐squared analysis. The data were fit to the model log(*E*(*Y_i_*)) = log *t_i_* + *βx_i_*, where *E*(*Y_i_*) is the expected value for the *i*th observation, *β* is the vector of regressions coefficients, *x_i_* is a vector of covariates for the *i*th observation, and *t_i_* is the offset variable used to account for differences in observation count period (e.g., total pfu). The offset (e.g., natural log of pfu) was given a constant coefficient of 1.0 for each observation, and log‐linear relationships between mutant count and test article concentration were specified by a natural log link function. *Post hoc* custom contrasts based on the asymptotic chi‐square distribution of the likelihood ratio statistic were conducted to compare each dose group with the control. Should visual examination suggest the need to truncate the top dose, data were re‐fit without the top dose, and if this resulted in an improved chi‐squared value, then the truncated results were retained, and this was used for potency determination (see below).

The potency of the responses for each significant tissue/endpoint combination was determined using ordinary least‐squares linear regression and expressed as the slope of the linear portion of the dose–response function. Individual animal genotoxicity results are available from the author upon request.

## RESULTS

### Analysis of Sealcoat and Sealcoat Extract

Chemical analysis of the CT‐based sealcoat revealed that there are 44.2 g (4.4% w/w) of the US EPA priority PAHs per kg of sealcoat, including 8.66 g (0.87% w/w) of US EPA class B2 PAHs (i.e., probable human carcinogens), and 1.91 g (0.19% w/w) of benzo(*a*)pyrene (BaP) (Table 1). Concentrations in the sealcoat extract are expressed in terms of sealcoat equivalents (eqs), which were calculated based on the amount of crude sealcoat that was extracted and submitted for chemical analysis. Additionally, we calculated the total amount of PAHs present in a 15‐L pail of CT‐based sealcoat (i.e., the format available for retail purchase). The results indicate that a 15‐L pail contains more than 750 g of priority PAHs and 147 g of B2 carcinogenic PAHs, including 32 g of BaP (Table 1).

**Table 1:** Concentrations of Polycyclic Aromatic Hydrocarbons (PAHs) in CT‐Based Driveway Sealcoat Extract (Expressed Per Unit Weight of Crude Sealcoat), as Well as the Total Amount of PAHs per 15‐L pail (i.e., the Commercially Available Size)

Polycyclic aromatic hydrocarbons	CAS #	Sealcoat (mg PAH/kg sealcoat)	Per 15‐L pail (g)
Acenaphthene^a^	83‐32‐9	2,433	41.30
Acenaphthylene^a^	208‐96‐8	BDL	N/A
Anthracenea	120‐12‐7	1,582	26.85
Benz[*a*]anthracene^a,b^	53‐55‐3	1,325	22.50
Benzo[*a*]pyrene^a,b^	50‐32‐8	1,906	32.35
Benzo[*b*]fluoranthene^a,b^	205‐99‐2	1,407	23.89
Benzo[*g,h,i*]perylene^a^	191‐24‐2	958	16.26
Benzo[*k*]fluoranthene^a,b^	207‐08‐9	784	13.30
Biphenyl	92‐52‐4	87	1.48
Chrysene^a,b^	218‐01‐9	2,409	40.89
Dibenz[*a,h*]anthracene^a,b^	53‐70‐3	151	2.56
Fluoranthene^a^	206‐44‐0	8,706	147.78
Fluorenea	86‐73‐7	1,867	31.69
Indeno[1,2,3‐*cd*]pyrene^a,b^	193‐39‐5	682	11.58
1‐Methylnaphthalene	90‐12‐0	201	3.42
2‐Methylnaphthalene	91‐57‐6	351	5.96
Naphthalene^a^	91‐20‐3	725	12.32
Phenanthrene^a^	85‐01‐8	11,850	201.15
Pyrene^a^	129‐00‐0	6,820	115.76
**Total**		**44,242**	**751.01**
**Total 16 US EPA priority pollutant PAHs**		**43,603**	**740.16**
**Total US EPA B2 carcinogenic PAHs**		**8,663**	**147.06**

The chemical abstract service number (i.e., CAS #) for each PAH is provided.

Designated a US EPA priority pollutant.

Designates a US EPA B2 carcinogen; eq: equivalents; BDL: below detection limit.

### DNA Adduct Frequency

**Figure 1:**
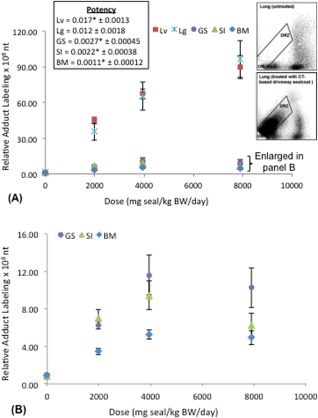
Relative adduct labeling [DNA adducts per 10^8^ nucleotides (nt)] in tissues from Muta™Mouse orally exposed to CT‐based driveway sealcoat (seal). Total frequency of adducts was determined from the diagonal radioactive zone (DRZ). **A**: Dose response–data and potency (i.e., the slope of the linear portion of the dose–response function) is presented for each tissue. Lv: liver, Lg: lung, GS: glandular stomach, SI: small intestine, BM: bone marrow. * indicates where data were truncated during statistical analysis, therefore the mutagenic potency is a result of fewer than 4 dose groups. **B**: Restricted *y*‐axis scale to better display dose‐response data for GS, SI, and BM. *Inserts*: Representative autoradiographic profiles of DNA adducts in lungs from untreated mice or mice subchronically exposed to CT‐based driveway sealcoat (these profiles are representative of adduct profiles obtained with DNA from other mouse tissues including liver, glandular stomach, small intestine, and bone marrow) . Solvent conditions for the separation of PAH‐derived DNA adducts using thin‐layer chromatography were as follows: D1, 1.0M sodium phosphate, pH 6.0; D3, 3.5M lithium‐formate, 8.5M urea, pH 3.5; D4, 0.8M lithium chloride, 0.5M Tris, 8.5M urea. The origins (OR), at the bottom left corner, were cut off before imaging.

Bulky DNA adduct frequency, determined using ^32^P‐postlabeling analyses, was employed to document tissue‐specific exposures to DNA‐damaging agents in the administered sealcoat extract. The results revealed statistically significant increases in DNA adduct frequency relative to controls in all tissues examined (Fig. 1). Furthermore, damage frequency increased in a dose‐dependent manner, with potency (i.e., magnitude of response increase per unit dose increase) varying across the tissues examined. Analyses of the dose–response relationships revealed the most potent response (i.e., highest slope) for the liver (maximum response up to 102‐fold over control), followed by the lung (maximum response up to 60‐fold over control), glandular stomach (maximum response up to 14‐fold over control), small intestine (maximum response up to 8‐fold over control), and finally the bone marrow (maximum response up to 5‐fold over control).

### *lacZ* Mutant Frequency

**Figure 2:**
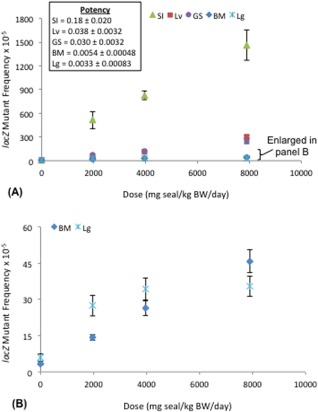
The *lacZ* mutant frequency in tissues from Muta™Mouse orally exposed to CT‐based driveway sealcoat (seal). **A** Dose–response data and potency (i.e., the slope of the linear portion of the dose–response function) is presented for each tissue. SI: small intestine, Lv: liver, GS: glandular stomach, BM: bone marrow, Lg: lung. **B** Restricted *y* axis to better display dose–response data for BM and Lg.

The frequency of *lacZ* mutants was assessed in five tissues. The results revealed a statistically significant response for each tissue with dose‐dependent increases in mutant frequency relative to controls and variations in potency across the tissues examined (Fig. 2). The most potent response (i.e., highest slope) was observed in the small intestine (maximum response up to 154‐fold above control), followed by the liver (maximum response up to 51‐fold above control), glandular stomach (maximum response up to17‐fold above control), bone marrow (maximum response up to 14‐fold above control), and finally lung (maximum response up to 6‐fold above control). The fact that the highest responses were seen in the site‐of‐contact and related tissues emphasizes the importance of examining these tissues following an oral administration.

### Micronucleus Frequency

**Figure 3:**
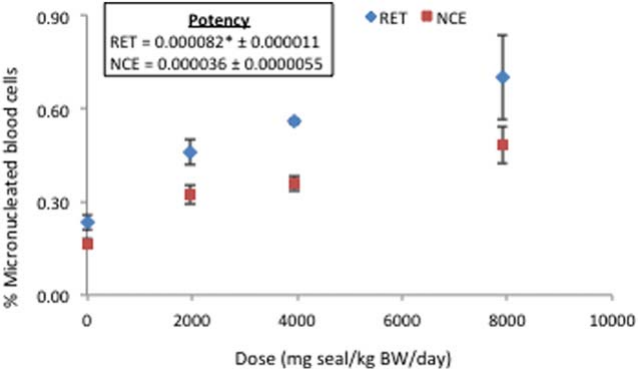
The frequency of micronuclei in peripheral blood from Muta™Mouse orally exposed to CT‐based driveway sealcoat (seal). Potency (i.e., the slope of the linear portion of the dose‐response function) is presented for each tissue. RET: reticulocytes, NCE: normochromatic erythrocytes. * indicates where data were truncated during statistical analysis, therefore the mutagenic potency is a result of fewer than four dose groups.

The frequency of micronucleated red blood cells was assessed as a measure of clastogenicity in hematopoietic tissue. Statistically significant increases in micronucleated RETs and NCEs were observed relative to controls, with a 2.3‐fold greater potency in RETs (i.e., a potency of 0.000082 in RETs versus 0.000036 in NCE) (Fig. 3). The results emphasize the importance of scoring RETs to examine the effects of recent damage.

## DISCUSSION

This study used a 28‐day repeat‐dose oral exposure of transgenic Muta™Mouse to assess the multiorgan genotoxicity of a PAH‐containing extract of a commercially available CT‐based driveway sealcoat. The results obtained clearly indicate that oral exposure to the purified extract induces both significant increases in DNA damage (i.e., adducts) and mutations (i.e., *lacZ*) across several tissues, as well as chromosome damage (i.e., MN) in peripheral blood cells.

Chemical analysis of the CT‐based sealcoat examined here revealed that it contains over 4% PAHs by weight, with approximately 0.9% US EPA B2 PAHs. In comparison with available information on other CT‐based sealcoats, the product examined in this study contained a lower concentration of PAHs. For example, in a study by Bryer et al. [2006], the sealcoat contained 23% PAHs by weight, with 5% B2 PAHs. Bommarito et al. [2010] reported that the CT‐based sealcoat examined in their study was approximately 8% PAHs by weight, and nearly 3% B2 PAHs. It is important to note that crude CT, CT pitch and products derived from CT pitch contain complex mixtures of PAHs, with their relative composition concentration varying widely with source and type of material (e.g., crude CT versus high‐temperature pitch). Additionally, sealcoats such as that examined here can contain 10–35% CT by weight and the final PAH content of the product will depend on the CT concentration in each batch. Although the PAH content of CTs will vary according to the type of material (i.e., crude or refined) and its source, the material generally used for the production of CT‐based pavement sealants is a derivative of CT pitch known as RT‐12. Although one might expect some consistency in the PAH content of CT‐based sealcoat products containing RT‐12, the source of the crude CT and the exact RT‐12 concentration in each sealcoat batch (i.e., 10‐35% by weight) affects the final PAH content of the sealcoat [USGS, 2011]. Moreover, the certificate of analysis for the National Institute of Standards and Technology Standard Reference Material 1597 (i.e., a complex mixture of PAHs from CT), which employed the Wise et al. [1988] method also used here, notes the presence of other polyaromatic compounds including sulphur heterocyclics, along with PAHs. This is consistent with our earlier work [Lundstedt et al., 2006; Lemieux et al., 2008] that employed a similar approach to prepare PAH‐containing extracts from CT‐contaminated soils, and noted that extracts can contain *N*‐ and *S*‐heterocyclics, in addition to homocyclic PAHs, all of which may contribute to the overall genotoxicity of the mixture.

The most potent DNA adduct response was observed in liver and lung, followed by glandular stomach. This pattern of CT‐induced DNA adducts across murine tissues following oral exposure is similar to what was observed in the CT study of Culp and Beland [1994]. More specifically Culp and Beland assessed bulky DNA adduct frequency in several mouse tissues following dietary administration of CT for 21 days, and noted that levels were highest in lung, followed by liver and forestomach [Culp and Beland, 1994]. The presence of stable DNA adducts in a particular tissue indicates that the tissue was exposed to DNA‐reactive metabolites (e.g., PAH‐diol‐epoxides). This can occur via CYP1‐mediated metabolism within the target tissue, and/or alternatively, metabolism in the gastrointestinal system followed by systemic circulation of reactive metabolites and tissue delivery. For example, the reactive form of BaP (i.e., benzo(*a*)pyrene‐diol‐epoxide; BPDE) has been identified in serum following an oral exposure to BaP [Ginsberg and Atherholt, 1989]; however, due to the highly reactive nature of these metabolites, it is more likely that the production of DNA‐reactive metabolites occurs *in situ* in each tissue. Indeed, it has previously been demonstrated in several mouse models that the tissues examined here have constitutive or inducible levels of CYP1A1, 1A2, and/or 1B1, all of which are known to convert PAHs to DNA‐reactive metabolites [Choudhary et al., 2003; Nebert et al., 2004; Uno et al., 2008; Renaud et al., 2011; Nebert et al., 2013; Arlt et al., 2015]. Moreover, our lab group has previously demonstrated induction of CYP1A1 and 1B1 in Muta™Mouse lung and liver following an identical oral exposure regime to several individual PAHs [Labib et al., 2015].

The most potent response for induction of *lacZ* mutations was observed in the small intestine, followed by liver, glandular stomach, bone marrow, and lung. For an oral exposure route, glandular stomach and small intestine are both initial site‐of‐contact tissues, with liver being closely related; whereas, both bone marrow and lung require distribution of the sealcoat compounds and/or compound metabolites via systemic and/or pulmonary circulation. These results support the contention that the production of DNA‐reactive metabolites occurs *in situ*. It is therefore not unexpected that the highest level of response induction was observed for site‐of‐contact and related tissues (i.e., stomach, liver, small intestine) prior to complete metabolism, detoxification and excretion. Nebert et al. [2013] demonstrated the importance of metabolism in small intestine and liver in the initial detoxification of orally‐delivered BaP. Efficient detoxification in the gastrointestinal system, and subsequent excretion of conjugates via the bile, would reduce the amount of compound and/or compound metabolite available for delivery to distal tissues. This contention is consistent with the high levels of CYP1 isozymes that have been observed in liver and small intestine (i.e., CYP1A2 in the liver, CYP1A1/1B1 in small intestine) [Uno et al., 2008; Shi et al., 2010a,b]. These isozymes are capable of converting PAHs into DNA‐reactive metabolites, thus contributing to elevated mutagenic responses in these tissues.

Despite significant increases in genotoxic damage across all endpoints and tissues, the results show clear differences in the tissue‐specific patterns of DNA adduct and *lacZ* mutation induction. As noted, the most potent adduct responses were observed in liver and lung; whereas, for *lacZ* mutations, the most potent response was observed for small intestine, with the least potent response observed in lung. Although the precise cause of this discrepancy remains to be determined, it seems reasonable to assert it relates to tissue‐specific differences in repair capacity and cellular proliferation rate. Conversion of DNA damage to permanent sequence changes (i.e., mutations) is controlled by a complex series of dynamic processes that control damage processing and repair, and cellular proliferation, and although a discussion of the mechanistic nuances of the process is beyond the scope of this work, it is interesting to note that cells in the lung and liver have relatively low proliferation rates in comparison with the other tissues examined herein (unpublished data). This would be expected to limit the conversion of adducts to mutations; moreover, tissue‐specific differences in damage processing and repair can be expected to augment or diminish the establishment of mutations. Although most cells in liver and lung tissues have low mitotic indices, lung has been shown to have an elevated capacity for error‐free lesion bypass that could contribute to a reduction in the conversion of adducts to mutations [Velasco‐Miguel et al., 2003]. More specifically, Ogi et al. [2002] noted that expression of the translesion DNA polymerase Pol κ is AhR‐dependant (i.e., PAH‐inducible), and Bi et al. [2005] noted that Pol κ is required for recovery from BPDE‐induced cell cycle checkpoint. Thus, although the precise causes of the observed crosstissue patterns in induced DNA adduct and mutation levels remain to be determined, dynamic differences in tissue specific metabolism, cellular growth, and DNA damage and repair likely play key roles.

Exposure to the sealcoat extract induced a significant increase in the frequency of micronucleated RETs and NCEs, although the fold‐increase in event frequency is much lower in comparison with the *lacZ* mutation and DNA adduct endpoints. It is well recognized that the primary mode of action for genotoxic PAHs is induction of mutations via formation and mis‐repair of bulky adducts; however, PAHs have also been previously shown to induce clastogenic effects and elevated levels of chromosomal damage [Glatt et al., 1990; He and Baker, 1991; Crofton Sleigh et al., 1993; Warshawsky et al., 1995; Whong et al., 1994; Nishikawa et al., 2005; Schober et al., 2006; Lemieux et al., 2011; Abramsson Zetterberg et al., 2013]. The results obtained showed significant elevations in micronuclei, which are thought to be produced during cellular replication following DNA double strand breaks [Fenech et al., 2011]. These results are consistent with a previous study by our group that employed an identical study design to evaluate the genotoxicity of 8 priority PAHs. The results of that study showed that 4 of the priority PAHs examined (i.e., BaP, dibenz(*a,h*)anthracene, benzo(*b*)fluoranthene, and benzo(*k*)fluoranthene) induced significant increases in the frequency of micronucleated red blood cells [Long et al., 2016].

The product instructions for the sealcoat examined in this study indicates that a 15‐L pail contains sufficient material to cover an area of 20 to 49 m^2^, and additionally, that it is a “1‐year sealcoat” that should be reapplied annually. Thus, for homeowners with a typical double‐car driveway (e.g., ∼36 m^2^) at least a full pail of sealcoat would be required for annual pavement maintenance, and therefore annual application of total priority PAHs for a typical household would exceed 750 g, including 147 g of US EPA B2 carcinogenic PAHs and 32 g of BaP. Despite the level of PAHs in coal tar‐based sealcoat, genotoxic hazard, and the recommended application amount and frequency, the risk of adverse human health effect depends on the potential for exposure, as well as the route, level, and duration of exposure. Clearly, the magnitude of human exposure to the product, and by extension the PAHs in the product, will depend on the magnitude and frequency of contact with the product (e.g., during application), contact with the pavement surface, and contact and/or oral ingestion of PAH‐contaminated particulate material (e.g., pavement dust and house dust) from weathered pavement. Although currently available information does not permit accurate determination of total human exposure to PAHs in the product or materials derived from the product, studies such as that by Williams et al. [2013] estimated the level of exposure (in ng BaP equivalents/kg BW/day) of individuals inhabiting residences adjacent to surfaces treated with CT‐based sealcoats. Moreover, Williams et al. estimated the excess lifetime cancer risk associated with nondietary ingestion of PAH‐contaminated soils and SHDs collected in close proximity to CT‐sealed pavements, and determined that values can exceed 1 × 10^−4^ [Williams et al., 2013], a level that exceeds the essentially negligible cancer risk for contaminated site risk assessment [Health Canada, 2012].

Published studies that investigated the murine carcinogenicity of CT have examined crude high‐temperature CT rather than the aforementioned refined material that is used in the production of CT‐based sealcoat products [Culp et al., 1998; Goldstein et al., 1998]. Nonetheless, it is interesting to compare the results of the Culp et al. [1998] and Goldstein et al. [1998] studies to those obtained here. A 2‐year rodent cancer bioassay involving B6C3F1 mice fed CT‐amended food found carcinomas in liver, lung, forestomach, and small intestine, with the highest tumour incidence observed in small intestine followed by lung [Culp et al., 1998; Goldstein et al., 1998]. This tumour induction pattern corresponds with the results of this study (i.e., highest levels of mutations and/or adducts in lung, liver and intestine). Despite the facts that not all mutagens are necessarily carcinogens and that the current study examined an extract of a CT‐containing pavement sealcoat rather than crude CT, the cross‐tissue, multi‐endpoint responses observed herein, and the correspondence between genotoxicity herein and earlier carcinogenicity results, strongly suggest that oral exposure to CT‐based sealcoat products would also induce rodent carcinomas in multiple tissues in longer‐term studies. This is especially concerning, since numerous studies by Mahler, van Metre, and colleagues [Metre et al., 2008; Mahler et al., 2010; Van Metre et al., 2012; Williams et al., 2013] have clearly demonstrated a potential for human exposure to mutagenic carcinogens in environmental samples (e.g., air, SHD, soil) collected in the vicinity of pavements treated with CT‐based sealcoats.

## CONCLUSION

CT‐based sealcoat products contain high levels of priority PAHs, including several mutagenic carcinogens, and previous work has demonstrated that environmental samples derived from sealed pavements (e.g., runoff water) or collected in the vicinity of surfaces treated with CT‐based sealcoats are similarly contaminated with priority PAHs and induce toxic/genotoxic effects in a range of organisms. Moreover, earlier work has documented the potential for human exposure via contact with PAH‐contaminated environmental matrices such as soil and SHD collected near surfaces treated with CT‐based sealcoats. The results obtained in this study clearly demonstrate that CT‐based pavement sealcoat extract has the ability to induce significant increases in genetic damage, including DNA adducts, mutations and chromosomal damage, in several tissues of orally exposed mice. Although it is not possible to quantitatively translate the magnitude of the observed rodent responses into effects that may occur in humans, the work provides conclusive identification of *in vivo* mammalian genotoxic hazard from oral exposure to a PAH extract from coal tar‐based driveway sealcoat.

## AUTHOR CONTRIBUTIONS

Dr. Paul White, Dr. Volker Arlt, and Ms. Alexandra Long designed the study and secured the funding. Ms. Long extracted the sealcoat, coordinated animal exposures and tissue processing, conducted DNA extractions, and analysed the data. Ms. Margaret Watson conducted DNA extractions and scored the frequency of *lacZ* mutants. Dr. Arlt conducted the analysis of DNA adducts. Ms. Long prepared the manuscript with input from all authors. All authors approved of the final version of the manuscript.
